# Targeting p16-induced senescence prevents cigarette smoke-induced emphysema by promoting IGF1/Akt1 signaling in mice

**DOI:** 10.1038/s42003-019-0532-1

**Published:** 2019-08-09

**Authors:** Christopher T. Cottage, Norman Peterson, Jennifer Kearley, Aaron Berlin, Ximing Xiong, Anna Huntley, Weiguang Zhao, Charles Brown, Annik Migneault, Kamelia Zerrouki, Gerald Criner, Roland Kolbeck, Jane Connor, Raphael Lemaire

**Affiliations:** 1 Research and Early Development, Respiratory, Inflammation and Autoimmune (RIA), BioPharmaceuticals R&D, AstraZeneca, Gaithersburg, MD 20878 United States; 2 Temple Lung Center, Philadelphia, PA 19140 USA

**Keywords:** Molecular biology, Senescence, Stem cells

## Abstract

Senescence is a mechanism associated with aging that alters tissue regeneration by depleting the stem cell pool. Chronic obstructive pulmonary disease (COPD) displays hallmarks of senescence, including a diminished stem cell population. DNA damage from cigarette smoke (CS) induces senescence via the p16 pathway. This study evaluated the contribution of p16 to CS-associated lung pathologies. p16 expression was prominent in human COPD lungs compared with normal subjects. CS induces impaired pulmonary function, emphysema, and increased alveolar epithelial cell (AECII) senescence in wild-type mice, whereas CS-exposed p16^−/−^ mice exhibit normal pulmonary function, reduced emphysema, diminished AECII senescence, and increased pro-growth IGF1 signaling, suggesting that improved lung function in p16^−/−^ mice was due to increased alveolar progenitor cell proliferation. In conclusion, our study suggests that targeting senescence may facilitate alveolar regeneration in COPD emphysema by promoting IGF1 proliferative signaling.

## Abstract (web summary)

Cottage et al. show that *p16* deficiency promotes the proliferation of alveolar progenitor cells, improving the lung function in mice. This study suggests that targeting senescence pathways may facilitate alveolar regeneration in chronic obstructive pulmonary diseases by activating cell-proliferation signals through insulin-like growth factor 1.

## Introduction

Chronic obstructive pulmonary disease (COPD) is the third leading cause of death in the United States^1^ and is associated with a compromised quality of life. The economic burden associated with the disease is substantial. Characteristics of COPD include inflammation^2^, tissue remodeling, and emphysematous alveolar destruction^3^, leading to enlarged air spaces with less surface area capable of gas exchange^4,5^. Lung exposure to contaminants and pollutants are risk factors for COPD, including cigarette smoking (CS)^6^. Aside from smoking cessation, no therapeutic intervention has been identified and research continues to investigate the molecular mechanisms driving disease progression. Many of the pathological processes identified in COPD are mediated by CS, including altered homeostatic apoptosis proliferation, production of extracellular matrix (ECM)-degrading proteases and oxidative stresses, as well as telomere dysfunction, leading to the activation of the DNA damage response pathway and ultimately cellular senescence^7–9^. Senescent cells produce and secrete numerous harmful pro-inflammatory and degrading mediators, collectively called the senescence-associated secretory phenotype (SASP). SASP proteins have been shown to be upregulated in pathologies related to accelerated aging^10^ and are known to perpetuate inflammation and tissue remodeling in COPD^11–13^. Development of effective therapeutics to combat senescent cells may provide clinical benefit.

A universal marker for cell senescence does not exist but most senescent cells express p16 (p16^ink4A^), a cell cycle inhibitor that targets cyclin-dependent kinases (CDKs) and is important in wound-healing and tumor suppression^10,13–17^. Removal of p16+ senescent cells has been shown to be an efficient way of extending healthspan and reversing senescence-associated pathologies^18–20^. In transgenic mice overexpressing p16, cell proliferation is dramatically reduced and tissue regeneration is accordingly diminished, similar to that of an aged mouse^21^. Age-related replicative and regenerative signaling loss correlates with the diminution of the insulin/insulin-like growth factor (IGF1) pathway^22^. The IGF1 pathway plays a central role in various phases of the cell cycle, including proliferation, survival, and differentiation^23^. IGF1 asserts these many mechanisms through downstream Akt activity^24^. The serine/threonine kinase known as Akt regulates cell survival and proliferation in the lung through phosphorylation of several anti-apoptotic proteins, but can also stimulate proliferation by promoting cyclin D accumulation^25,26^. Cyclin D progresses the cell cycle from G1 to S by binding to CDK4/6 and phosphorylating retinoblastoma^27^. By binding to cyclin D, p16 prevents cell cycle progression and proliferation^28^.

In the current study, we hypothesized that p16 plays a role in the pathological processes associated with smoking and COPD, and that deletion of p16 protects the lung from the development of emphysematous-like tissue remodeling. We examined human lung tissue from COPD patients and normal control subjects, and found a substantial increase in p16-expressing alveolar cells in COPD patients. Using a transgenic mouse deficient for p16, we demonstrated that lungs of mice lacking p16 were structurally and functionally resistant to CS-induced emphysema due to activation of IGF1/Akt regenerative and protective signaling.

## Results

### p16 expression is increased in human COPD lungs

**Fig. 1:**
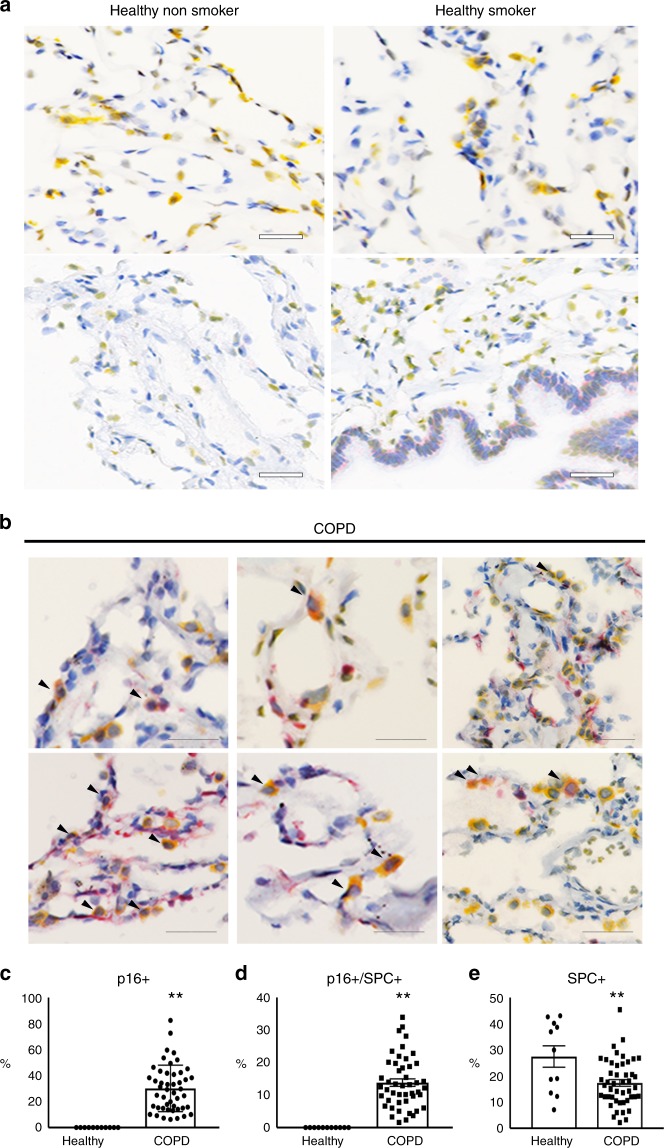
Immunohistochemical localization of p16 in human lungs. p16 (red) and SPC (yellow) staining in **a** healthy non-smoker, healthy smoker, and **b** COPD human lungs. Arrowheads indicate co-localization of p16 and SPC (scale bar = 40 µm). **c** Percentage of p16 + (**p < 0.0001), **d** p16 + /SPC + (**p < 0.0001), and **e** SPC + cells in healthy and COPD lungs (*p = 0.0034). *N* = 3–11

To assess the expression and localization of p16 protein in human COPD/emphysema, IHC was carried out on lung biopsies from age-matched patients diagnosed with emphysema, normal non-smokers, and normal (non-diseased) smokers. Supplementary Table 1 displays patient clinical data. Sections from normal non-smokers had little p16 staining, whereas normal smokers had low sporadic expression (p16 in red, Fig. 1a). COPD patients showed high expression of p16 across the lung, including bronchial and alveolar epithelia, and interstitial cells (Fig. 1b, c). Senescence was assessed in alveolar type II cells (AECII) by double IHC staining of p16 and SPC. We found that p16 is expressed in ~13.8% of SPC + alveolar type II cells (Fig. 1d), consistent with a reduced AECII population in COPD lungs (Fig. 1e) and suggesting a role of senescence in impaired alveolar regeneration in COPD lung. Senescence was also assessed in endothelial cells by p16 and CD31 IHC staining on serial sections. p16 rarely co-localized with CD31, suggesting senescent endothelial cells are not dominant contributors to COPD pathology (Supplementary Fig. 1).

### p16 deletion blocks the pathophysiological changes associated with cigarette smoke

**Fig. 2:**
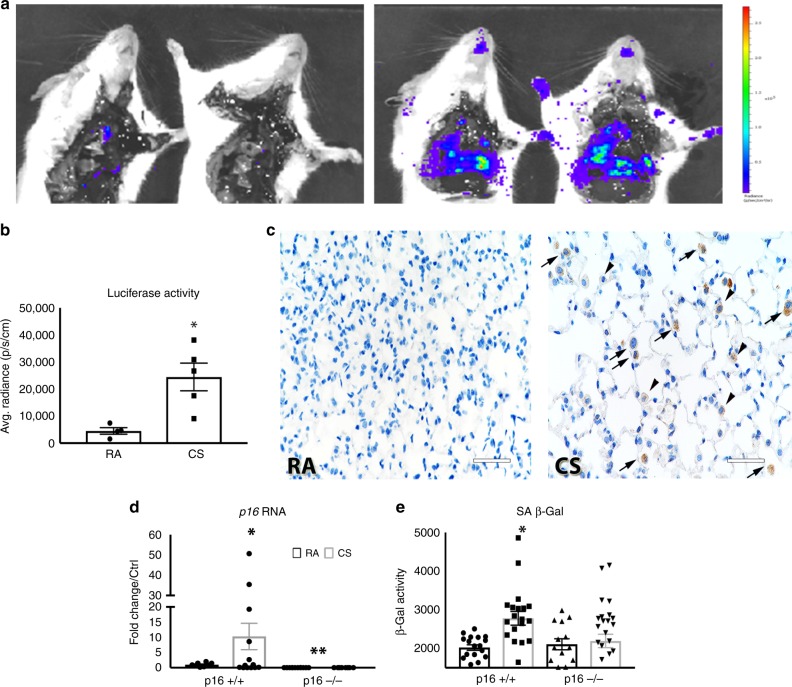
Cigarette smoke promotes p16 promoter-driven luciferase activity and senescence. **a** Luciferase imaging of p16^−/−^ lungs from mice exposed to RA or CS for 4 months. **b** Average radiance measuring luciferase activity in p16^−/−^ lungs (*p = 0.0114). **c** Luciferase staining in RA- and CS-treated lungs; images were taken of alveolar space in p16^−/−^ mice. Arrowheads indicate luciferase-positive AECII cells and arrows indicate macrophages (scale bar = 40 µm). **d** Quantitative real-time PCR of *p16* RNA (*p = 0.0307, **p = 0.0003) and **e** SA β-Gal activity measured in whole lung homogenates after 4 months of RA or CS (*p = 0.0005). *N*, p16^+/+^ RA = 4–8, p16^+/+^ CS = 10–14, p16^−/−^ RA = 4–7, and p16^−/−^ CS = 12–14 mice

In order to evaluate the role of the p16-mediated senescence pathway, we utilized a transgenic mouse in which firefly luciferase protein is knocked in downstream of the p16 promoter, functionally knocking out p16 expression (p16^−/−^)^28^. Wild-type (p16^+/+^) and p16^−/−^ mice were exposed to cigarette smoke (CS) for 4 months. Ex vivo isolation and imaging of the lungs was carried out (Fig. 2a). CS exposure induced a fivefold increase in p16 promoter-driven luciferase activity compared with room air (RA, Fig. 2b). In addition to bioluminescence, luciferase expression was detected by IHC. Minimal luciferase was expressed in RA lungs, yet luciferase staining was detectable in CS-treated lungs in multiple cell types, including AECIIs, macrophages, and interstitial cells (Fig. 2c). *p16* RNA increased ~10-fold in p16^+/+^ mice upon CS exposure (Fig. 2d). PCR analysis confirmed that p16^−/−^ lungs do not express p16 with or without CS. Concomitant to p16 expression, senescence-associated β-gal activity increased in p16^+/+^ lungs treated with CS; this induction did not take place in p16^−/−^ lungs (Fig. 2e).

**Fig. 3:**
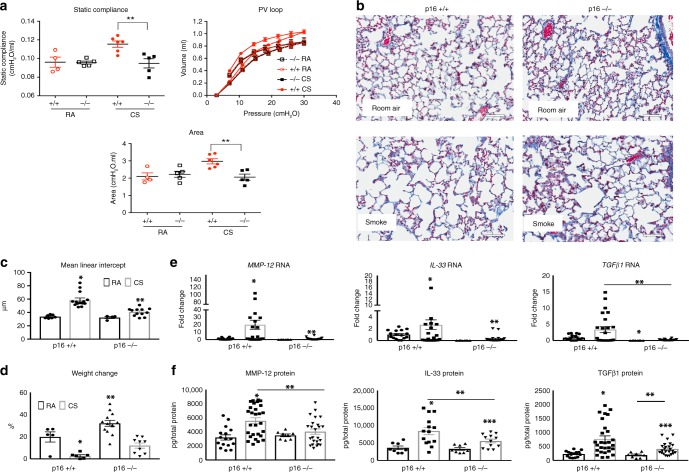
p16^−/−^ lungs maintain function and structure when challenged with CS. **a** Lung compliance (**p < 0.0177), pressure/volume, and area (**p < 0.0001) between the inflation and deflation limb of the PV loop measured using Flexi-Vent. **b** Representative Masson’s trichrome staining of lungs exposed to RA or CS (scale bar = 40 µm), mean linear intercept was determined **c** from these images and quantified (*p < 0.0001 and **p < 0.0001 vs. p16^−/−^ RA). **d** Body weight increases over 4 months, values are the percentage increase from day 0 (*p = 0.0031, **p < 0.0001). Quantitative RT-PCR analysis **e** of MMP-12 (*p = 0.029 vs. p16^+/+^ RA, **p = 0.0077 vs. p16^+/+^ CS), IL-33 (*p = 0.012 vs. p16^+/+^ RA, **p = 0.045 vs. p16^+/+^ CS), and TGFβ1 (*p = 0.0021 vs. p16^+/+^ RA, **p = 0.0036). After 4 months of CS, all samples were normalized to mouse GAPDH. **f** MMP-12 (*p = 0.0004 vs. p16^+/+^ RA, **p = 0.0088 vs. p16^+/+^ CS), IL-33 (*p = 0.004 vs. p16^+/+^ RA, **p = 0.023 vs. p16^+/+^ CS, ***p = 0.0452 vs. p16^−/−^ RA), and TGFβ1 (*p = 0.0005 vs. p16^+/+^ RA, **p = 0.0097, ***p = 0.0036 vs. p16^+/+^ RA), protein levels determined by ELISA. All protein levels are normalized to total protein in the lysate. All data is expressed as mean ± SEM, *n* = 4–14 mice per group

Pulmonary structure and function were also examined after 4 months of CS. In vivo respiratory mechanics were evaluated using Flexi-vent. CS substantially increased static lung compliance, pressure/volume, and *P*/*V* area in p16^+/+^ lungs (Fig. 3a), consistent with pulmonary emphysema. These changes were not detected in p16^−/−^ CS lungs, which were comparable to RA-treated mice. CS-induced changes in pulmonary compliance typically reflect reductions in elastic recoil caused by destruction in the alveolar wall and loss of tissue^33^. Accordingly, histological analysis showed that CS exposure causes large emphysematous alveoli alterations in p16^+/+^ lungs. Deletion of p16 markedly reduced CS-induced alterations (Fig. 3b). MLI quantification validated histology observations (Fig. 3c). MLI in p16^+/+^ lungs exposed to CS were double that of RA-treated, whereas p16^−/−^ lungs exposed to CS showed minimal increases, suggesting a role of p16 in alveolar structure homeostasis. Unlike p16^+/+^ mice, CS minimally affected p16^−/−^ mice weight, as they continue to gain weight throughout exposure. However, this can be attributed to CS-associated nicotine appetite suppression and not emphysema^34^ (Fig. 3d).

Mediators known to be upregulated in COPD, including MMP-12, IL-33, and TGFβ1, increased in p16^+/+^ lungs at both the RNA (Fig. 3e) and protein (Fig. 3f) levels after 4 months of CS compared with RA. In contrast, CS-induced levels of these mediators were reduced in p16^−/−^ mice (Fig. 3e). Increases of IL-33 and TGFβ1 protein were observed in p16^−/−^ mice; however, the protein levels (Fig. 3f) were not as elevated as those of p16^+/+^ CS. These data suggest that p16 depletion prevents COPD-associated detrimental mediators from reaching pathological levels and leading to the structural and functional alterations associated with CS exposure.

### p16 deletion reduces CS-induced cytokine production in lungs

**Fig. 4:**
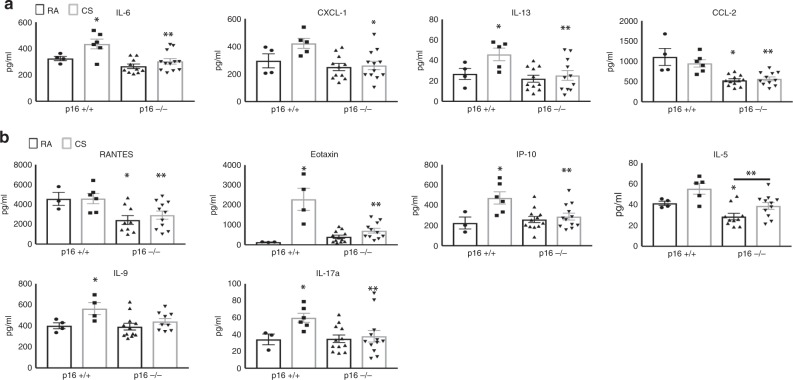
SASPs and Inflammatory cytokines upregulated with CS ameliorated in p16^−/−^ lungs. **a** Cytokines associated with senescent-associated secretory phenotype (SASP) and **b** inflammation on p16^+/+^ and p16^−/−^ lungs exposed to RA or CS. IL-6 *p = 0.0499, **p = 0.0043 vs. p16^+/+^ CS, CXCL-1 *p = 0.0084, IL-13 *p = 0.05, **p = 0.0260, CCL-2 *p = 0.0006 vs. p16^+/+^ RA **p = 0.0009 vs. p16^+/+^ CS, RANTES *p = 0.0346, **p = 0.0267 vs. p16^+/+^ CS, Eotaxin *p = 0.0221 vs. p16^+/+^ RA, **p = 0.0010 vs. p16^+/+^ CS, IP-10 *p = 0.0372 vs. p16^+/+^ RA, **p = 0.0108, IL-5 *p = 0.0308, **p = 0.0377, IL-9 *p = 0.0425, IL-17a *p = 0.0234, **p = 0.05 vs. p16^+/+^ CS. *N* = 4–12 lungs per group

Cytokines and growth factors typically associated with SASP and inflammation were measured by Luminex assays in lungs from RA- and CS-treated mice. SASP mediators, including IL-6, CXCL-1 (chemokine ligand-1), IL-13, and CCL-2 (chemokine ligand 2), were elevated in CS-treated p16^+/+^ mice (Fig. 4a). CS-induced cytokine levels in p16^−/−^ mice were comparable to those of p16^+/+^ RA-exposed. Similarly, inflammatory mediators, including RANTES, eotaxin, IP-10 (chemokine ligand 10), IL-5, IL-9, and IL-17a were all upregulated with CS in p16^+/+^ lungs, unlike in p16^−/−^ lungs where cytokine induction was prevented (Fig. 4b).

### p16 deletion reduces CS-induced senescence in AECIIs

**Fig. 5:**
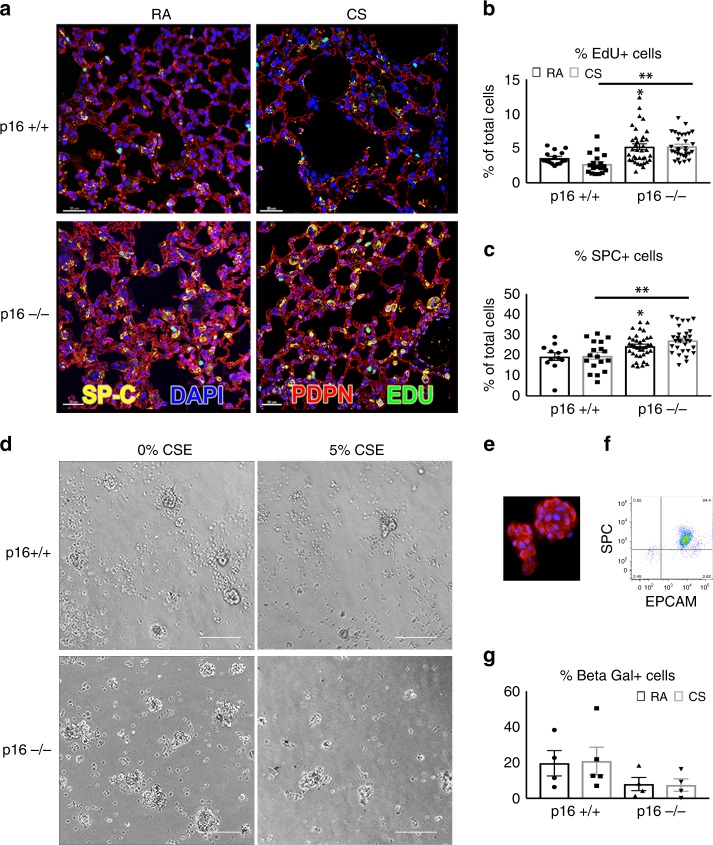
Increased cell proliferation and reduced AECII senescence in p16^−/−^ lungs. **a** Twenty-four hours post IP EdU injection (50 mg/kg), p16^+/+^ and p16^−/−^ lungs exposed to RA and CS were fixed and stained with Click-It EdU (green), SPC (yellow), Podoplatin (PDPN, red), and DAPI (blue, scale bar = 30 µm). **b** Quantification of lung proliferating (EdU+) cells in the alveolar airspace. At least 1500 nuclei per airspace were counted in each lung *p = 0.0197, **p < 0001. *N* = 4–12 lungs per group. **c** Percentage of SPC+ cells in the alveolar region of the lung *p = 0.015, **p = 0.0004. **d** Images of Isolated AECIIs from p16^+/+^ and p16^−/−^ lungs treated with 5% CSE in 10% serum for 24 h and probed for β-galactosidase activity using C12FDG substrate (scale bar = 400 µm). **e** AECIIs immunostained with SPC (red) and DAPI (blue). **f** Flow cytometry analysis of AECII measuring purity of isolation. *X* axis is measuring EPCAM for epithelial cell specificity and *y* axis is measuring SPC for AECII specificity. **g** AECIIs treated with 5% CSE and C12FDG, and quantified with flow cytometry. Data represent the average ± SEM of four to five independent experiments

In vivo cell proliferation was assessed by EdU incorporation. Deleting p16 significantly increased cell proliferation from 3.6% to 5.3% (Fig. 5a, b, *p* < 0.0197). CS exposure led to a reduction in proliferation in p16^+/+^ lungs from 3.6% to 2.7%, unlike the effect in p16^−/−^ lungs where proliferation was maintained (Fig. 5b). As AECIIs are essential to homeostasis and regeneration of alveolar epithelium, we assessed the number of these lung progenitors using IHC for SPC+. Consistent with previous studies, we found that p16^+/+^ lungs were 15–20% SPC + AECIIs throughout the alveolar region (Fig. 5c)^35^. In RA conditions, deletion of p16 resulted in an increase in the percentage of SPC+ cells from 19.23% to 24.32% (Fig. 5c, RA p16^+/+^ vs. RA p16^−/−^). CS exposure increased the numbers of SPC+ cells from 19.61% to 27.19%, supporting the observed protection from CS-mediated alveolar structure destruction. Consistent with the proliferative phenotype of p16^−/−^ mice, the cell cycle inhibitor gene *p21* was dramatically reduced in p16^−/−^ lungs compared with p16^+/+^ (Supplementary Fig. 2). In contrast, p16^+/+^ mice showed a threefold increase in *p21* upon CS exposure compared with RA, strongly suggesting a role of senescence in CS-induced pulmonary dysfunction observed in p16^+/+^ mice. To test whether AECIIs from p16^−/−^ lungs were indeed resistant to CS-induced senescence, we isolated AECIIs from mouse lungs. The purity of isolated AECIIs was >94%, as determined by epthelial cellular adhesion molecule (EPCAM) and SPC flow cytometry (Fig. 5e, f and Supplementary Fig. 3). AECIIs were cultured in a three-dimensional system and treated with 5% cigarette smoke extract (CSE). AECIIs in full media formed alveolar spheres demonstrated by SPC staining in Fig. 5d, e. CSE-treated AECIIs formed fewer spheres but were still viable (Fig. 5d). When exposed to CSE, the percentage of senescent p16^+/+^ AECIIs increased as assessed by flow β-gal activity (Fig. 5g). Deletion of p16 completely prevented the CSE-induced senescence of AECII (p16^+/+^ CS vs. p16^−/−^ CS).

### p16 deletion promotes proliferative signaling in the lung

**Fig. 6:**
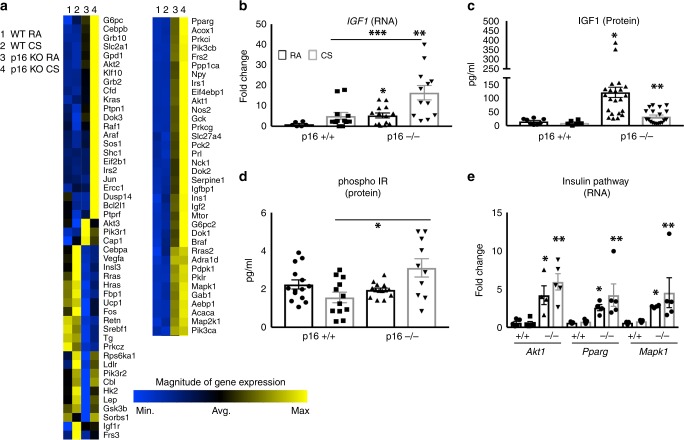
Increased IGF1 signaling in p16^−/−^ lungs. **a** Heat map of 84 insulin-related genes in lungs treated with CS. Genes in blue are reduced, while genes in yellow are increased. *N* = 5 lungs per group. **b** *IGF* RNA levels measured by TAQman qPCR. *p = 0.016, **p = 0.007, ***p = 0.008. **c** IGF1 measured by ELISA from whole lung lysates. *p = 0.0028, **p = 0.0421 vs. + /+ CS. **d** Phospho-insulin receptor measured by ELISA (*p = 0.0085). **e** Levels of key proliferation signaling genes *Akt1* (*p = 0.0137, **p = 0.0011), *Ppar*γ (*p = 0.0009, **p = 0.0392), and *MapK1* (*p < 0.0001, **p = 0.039) from profiler array. *N* = 5 lungs per group

**Fig. 7:**
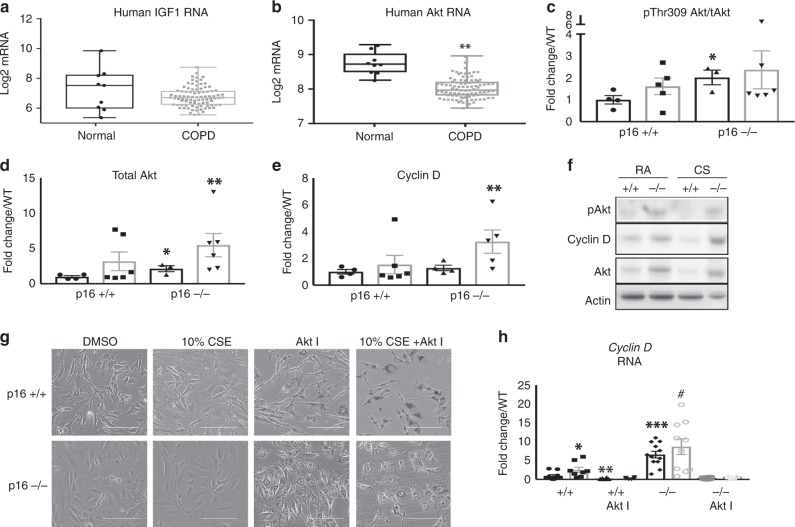
Diminished *IGF1* and *Akt* mRNA in human COPD lungs and loss of p16 increases Akt signaling in CS-exposed lungs. **a**, **b** Gene microarray analysis of whole lung lysates from 9 normal and 94 COPD donors **p = .85e^−6^. **c**–**e** Protein profile of phosphor-Akt Thr308 (*p = 0.0359), total Akt (*p = 0.0396, **p = 0.0416), and Cyclin D (**p = 0.0249). **f** Representative western blotting of whole lung lysates exposed to RA and CS (*n* = 4–6). **g** Representative images of fibroblasts isolated from p16^+/+^ and p16^−/−^ lungs then treated with DMSO or Akt I (10 μM, scale bar = 400 µm). **h** QPCR was performed to determine Cyclin D gene expression. Data are expressed as the average ± SEM of at least three independent experiments, *p = 0.0471, **p = 0.0230, ***p < 0.0001, #*p* = 0.001

To further investigate the mechanism by which p16^−/−^ mice are resistant to CS-induced emphysema, we performed array-based pathway-specific transcriptomic analysis. We found that members of the insulin pathway were uniquely upregulated in p16^−/−^ lungs and altered further when treated with CS (Fig. 6a). To validate the array analysis, we measured *IGF1* mRNA and protein by PCR and ELISA, respectively. Deletion of p16 increased *IGF1* mRNA and protein (Fig. 6b RA). This translated to a 7.83-fold increase in IGF1 protein in p16^−/−^ lung compared with p16^+/+.^ When treated with CS smoke, p16^−/−^ lungs maintained elevated levels of IGF1 compared with p16^+/+^ (Fig. 6c). Discrepancies between *IGF1* RNA and protein are likely due to CS-induced proteasome functions^36^. Phosphorylated insulin receptor (IR), the receptor known to be activated by IGF1, was upregulated in p16^−/−^ lungs upon CS exposure, unlike in p16^+/+^ mice where it decreased (Fig. 6d). Downstream mediators of the insulin/IGF1 pathway were also increased in p16^−/−^ lungs compared with p16^+/+^ mice, including *Akt1*, *PPARγ*, and *MapK1* (Fig. 6e). These data suggest that the structural and functional protection in p16^−/−^ mice is mediated by the pro-growth/anti-apoptotic IGF1 pathway. This led us to investigate this pathway in human COPD lung samples obtained from patients undergoing lung resection (and therefore predominantly GOLD IV, Supplementary Table 2). Human *IGF1* and *Akt1* mRNA were both diminished in COPD lung (Fig. 7a, b). As cellular damage from CS has been shown to target Akt specifically^37^, we looked at Akt in p16^−/−^ lungs. Active Akt (pThr309) level was doubled in p16^−/−^ lungs and increased slightly with CS (Fig. 7c). Similarly, total Akt was substantially elevated in p16^−/−^ lungs and increased with CS (Fig. 7d). Active Akt leads to an accumulation of cyclin D^27,38^, a molecule necessary for S phase entry and the target of p16 to induce senescence^39^. Cyclin D levels were comparable in p16^−/−^ and p16^+/+^ mice with RA; however, there was a much larger increase in cyclin D in p16^−/−^ compared with p16^+/+^ lungs upon CS exposure, supporting the observation of enhanced cell proliferation and regeneration in p16^−/−^ lung (Fig. 7e, f, Supplementary Fig. 4). To confirm that the cyclin D increase in p16^−/−^ lung was Akt-dependent, lung fibroblasts were isolated from p16^+/+^ and p16^−/−^ lungs, and treated with an Akt inhibitor along with 10% CSE. Phase-contrast images (Fig. 7g) confirmed that neither 10% CSE or the Akt inhibitor were toxic but instead inhibited proliferation. CSE-induced cyclin D in p16^+/+^ fibroblasts and the Akt inhibitor inhibited CSE-induced cyclin D (Fig. 7h). Cyclin D levels were sixfold higher in p16^−/−^ fibroblasts than in p16^+/+^ fibroblasts, and slightly increased with CSE. The Akt inhibitor entirely eliminated cyclin D (Fig. 7h), confirming that the cyclin D upregulation in p16^−/−^ lung fibroblasts was Akt-dependent.

## Discussion

COPD can be characterized as a disease of accelerated pulmonary aging. COPD patients experience many of the hallmarks of aging, including the deterioration of lung compliance, alveolar destruction, inflammation, genetic instability, and cellular senescence^40^. CS is recognized as a major contributor to COPD pathologies, as it contains reactive oxygen species that cause epithelial injury and inflammation throughout the central and peripheral airways, and lung parenchyma^41^. CS-induced chronic epithelial injury causes impaired tissue regeneration due to dysfunctional homeostatic balance between key tissue remodeling mechanisms such as cellular proliferation and senescence^6,37,42,43^. In the lung, stressed or damaged alveolar type I (AECI) cells undergo apoptosis leading to attempted repair by AECII progenitor cells within the alveoli, but over time with repeated challenge this cell replacement system declines and the ability to repair weakens^44^. To better understand the kinetics of CS-induced senescence, we treated p16^+/+^ and p16^−/−^ mice with CS for 1 month and 2 months, respectively. At 1 month, we were able to see a significant increase in senescence-associated β-gal activity that slightly increased at 2 months vs. RA (Supplementary Fig. 5a, *p* = 0.0183). Interestingly, in p16^−/−^ mice, although β-gal activity increases upon CS exposure at 1 month, the protective effects become evident at 2 months, with decreased β-gal activity. In addition to β-gal activity, we also examined the kinetics of p16 protein at earlier time points. Similar to β-gal activity, p16 expression increased gradually with continued exposure to CS (Supplementary Fig. 5b).

Decreased lung compliance seen in COPD is caused by the destruction of alveolar spaces induced by the breakdown of extracellular proteins and exhausted progenitor cell compartments. Blocking alveolar destruction and/or creating an environment that facilitates alveolar regeneration could provide an important approach to treating COPD and emphysema. In this study, p16^−/−^ pulmonary cells proliferated at a higher rate than p16^+/+^ in normal air and in the presence of CS. Increased proliferation resulted in a larger population of progenitor AECII cells leading to limited alveolar destruction. Our study demonstrates that promotion of cell hyperplasticity, upon deletion of a cell cycle inhibitor, contributes to improved lung structure and function, and may represent a valuable strategy to treat COPD and emphysema.

In addition to the direct effects of p16 deletion on the increased ability of the alveolar progenitor pool to proliferate, protection from CS-induced pathological mediators appears to be conferred as well, as evidenced by the attenuated induction of proteolytic enzymes and inflammatory cytokines (Fig. 3e, f and Fig. 4). Macrophages, recruited to the lung by the damage resulting from CS exposure, are known to be a dominant source of ECM-degrading enzymes such as MMP-12^45,46^. Supplementary Fig. 6a and 6b show that exposure to CS leads to increased macrophage numbers at the earliest time point evaluated (1 month) in both p16^+/+^ and p16^−/−^ lungs and the numbers remain similarly elevated across all time points evaluated. Interestingly, although MMP-12 expression was elevated in the wild-type mice with CS, the p16^−/−^ mice are protected from this increase (Supplementary Fig. 6c), suggesting that the macrophages may not be the only source of the MMP-12. As senescence is associated with an activated cell phenotype, the reduced MMP-12 may be the result a decreased secretory phenotype of the cells lacking p16.

**Fig. 8:**
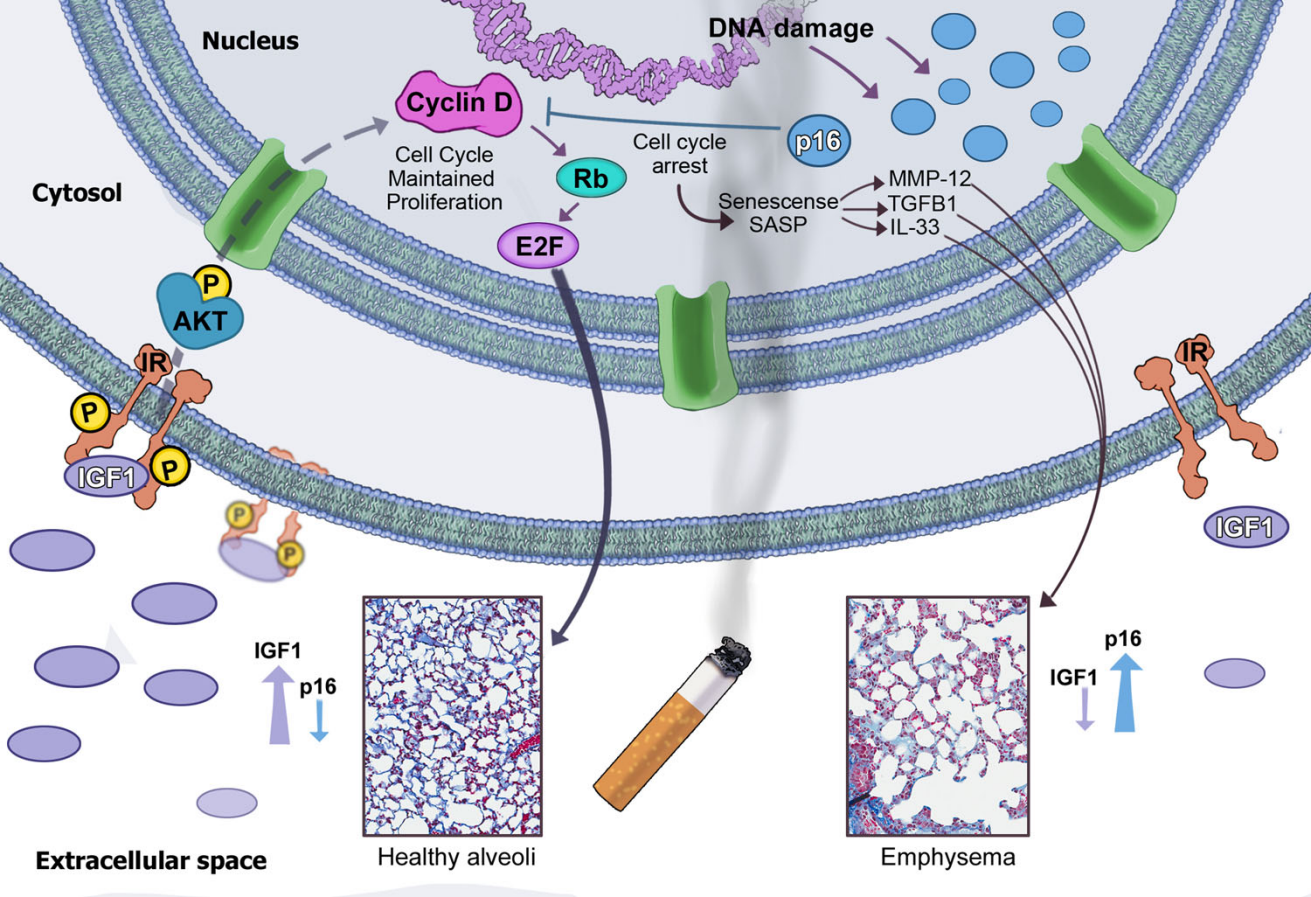
Proposed mechanism of protection. In the presence of chronic CS, p16 levels increase and cells become senescent. CS induces pro-inflammatory cytokines throughout the lung, and in the senescent cells SASPs are secreted, which perpetuate senescence and alveolar destruction (right side). When the cell cycle inhibitor p16 is not present (left side), basal levels of IGF1 are upregulated, resulting in stimulated insulin receptors and activation of Akt. Activated Akt stimulates cell proliferation evidenced by increases in Cyclin D expression and the maintenance of healthy alveoli

Our study reveals that levels of pro-growth mediators IGF1^47^ and Akt^25^ are diminished in COPD lung compared with normal (Fig. 7a, b), suggesting that a defective IGF1 pathway mediates, at least in part, the compromised tissue regeneration seen in COPD lungs. IGF1 is induced by growth hormone in the pituitary and is crucial in lung development and health^48^. Three IGF1 receptors IGF1R, IGF2R, and IR activate PI3 kinase and downstream Akt signaling to regulate metabolism, cell cycle, and apoptosis^23,49^. As depicted in Fig. 8, we propose that oxidant-containing CS induces DNA damage that activates the p16 senescence pathway. Senescent cells secrete SASP mediators, such as MMP-12, TGFβ1, and IL-33, affecting the extracellular matrix homeostasis, which in combination with the loss of regenerative capacity of senescent AECII progenitors, leads to alveolar destruction and emphysema. Inactivation of the p16 senescence pathway prevents CS-induced lung emphysema by upregulation of the IGF1 pathway with activation of Akt and cyclin D accumulation, promoting AECII proliferation and regeneration.

A recent report using a chronic (6 months) model of CS-induced COPD failed to find protective effects of p16 deletion^50^. There are important differences in our approach that likely account for the different outcomes. The p16^−/−^ transgenic mice used in their study have the entire Ink4a locus deleted, knocking down p16 and downstream p19, another tumor suppressor. With the entire locus knocked out, the mice spontaneously develop tumors in the spleen and liver, limiting the lifespan of the mice. The number of mice that survived CS exposure were extremely limited (3 of the 11), making conclusions very challenging if not impossible.

Considerable evidence exists linking p16 expression and a multitude of pathologies associated with aging, including cardiovascular disease^51^, osteoporosis^20^, diabetes^52^, age-related frailty^53^, and pulmonary disorders^10,43,54–57^. The emerging therapeutic approach of utilizing senolytics to remove senescent cells may provide benefit by leading to a more pro-resolution/regenerative cellular environment.

## Methods

### Animals

Wild-type B6(Cg)-Tyrc-2J/J (p16^+/+^) mice were obtained from The Jackson Laboratory; p16^Luc^ mice (Strain Code 01XBT -- B6.Cg-Cdkn2a ^tm3.1Nesh^ Tyr ^c-2J^/Nci) were obtained from the NCI mouse repository via Dr Norman Sharpless (University of North Carolina School of Medicine). Mice were housed and maintained in accordance with the Guide for Care and Use of Laboratory Animals and under the American Association for the Accreditation of Laboratory Animal Care I accreditation. All protocols used in these studies were approved by the Institutional Animal Care and Use Committee of MedImmune.

### Cigarette smoke exposure

p16^+/+^ and p16^−/−^ mice were exposed to cigarette smoke in a SIU48 machine (PromechLab AB, Vintrie, Sweden; 3R4F cigarettes from The Tobacco and Health Research Institute, University of Kentucky). Eight to 10-week-old female mice are exposed to 18 cigarettes twice a day, 5 days a week for 4 weeks, 8 weeks, and 16 weeks. The smoking period is 90 min per exposure with a 3 h rest period between exposures. In vivo Luciferase detection was performed on isoflurane-anesthetized mice following intraperitoneally injecting d-luciferin substrate (15 mg/ml in Dulbecco’s phosphate buffered saline (DPBS)).

### Histology

Human COPD and normal samples were purchased from Avaden BioSciences (Seattle, WA), Tissue Solutions (Glasgow, Scotland), and The National Resource Center (Philadelphia, PA). The study was approved by the Temple University Human Research Committee and all subjects provided their informed consent. All companies maintain informed consent from patients and maintain Institutional Review Board -approved protocols at every clinic with annual reviews. Surfactant Protein C (SPC, 1/1000, Millipore Sigma, AB3786), CD31 (0.80 µg/ml, Ventana ref #760–4378), and p16 (1 mg/ml, Ventana ref #705-4713) antibodies were used to stain 3 normal and 13 COPD human sections. To quantify expression, five random fields were analyzed per lung by Image J software.

Mouse lungs were perfused by instillation with Phosphate buffered saline (PBS) and PBS/ optimal cutting temperature compound (Fisher Healthcare, 23-730-571)-embedding solution, in order to ensure structural integrity, then fixed in 10% formalin. Then the lungs were embedded in paraffin and cut into 5 μm sections and Masson’s trichrome (MT) stain was performed using standard protocols. Determination of mean linear intercept (MLI), collagen deposition, and lung area were determined using Aperio Imagescope (Leica Biosystems, Buffalo Grove, IL) and Image J software (NIH). Specifically, for MLI, five random fields per lung were assessed on MT-stained lung sections. On average, 200 alveoli were recorded per lung and 4–14 lungs were measured per group. Immunohistochemistry (IHC) using antibodies against SPC (mouse 1/200, Millipore Sigma, AB3786), F4/80 (1/1000 CST, 70076), and Luciferase (1/8000. Abcam, ab21176) was performed using previously published protocols^29,30^. Briefly, slides were deparaffinized, then antigen retrieval was conducted using 10 mM sodium citrate. TNB blocking reagent (Perkin Elmer) was applied to reduce background signal as well as dilute primary and secondary antibodies. Chromogenic IHC stain was performed as above, then incubated with DAB Chromogen Solution (R&D Systems) and counterstained with hematoxylin. EdU was injected 24 h intraperitoneally prior to killing; incorporated EdU was imaged using the Click-it EdU Cell Proliferation assay (ThermoFisher, Carlsbad CA) as per the manufacturer’s protocol.

### AECII and fibroblast isolation

For alveolar epithelial cells (AECII) isolation, 8-week-old mice were killed, then Dispase and PBS were rabidly instilled through a cannula in the trachea. Following Dispase, 0.5 ml of warmed agarose was injected into the lung then covered with ice for 2 min. Lungs were then removed and incubated in 1 ml of Dispase for 45 min. After incubation, the cell suspension was filtered through progressively smaller cell strainers and nylon gauze as described previously^31^. Next, a discontinuous OptiPrep density gradient centrifugation step (Axis-Shield Alere Technologies Oslo, Norway) followed by centrifugation (130 × *g* for 8 min), then the cells were plated on 10 cm dishes that had been coated with CD45 and CD32 the previous day. After 2 h of incubation, the AECIIs are not bound to the plate and thus can be removed and cultured on top of 100% Matrigel (BD biosciences) in Dulbecco’s modified Eagle’s medium (DMEM, ThermoFisher). The cells that were bound to the 10 cm plate were collected and seeded in flasks containing DMEM supplemented with 10% fetal bovine serum, giving rise to pure fibroblast population after a week in culture.

### Real-time PCR reaction

RNA was isolated from whole lung lobes using a Zymo Research kit (R1065, Irvine, CA) according to their protocol. RNA was reverse transcribed using iScript Reverse Transcription Supermix (Bio-Rad, Hercules, CA) and TaqMan primers for indicated genes were purchased from ThermoFisher Scientific (Carlsbad, CA), *p16* (Mm00494449_m1), *MMP-12* (Mm00500554_m1), *interleukin (IL)-33* (Mm00505403_m1), *TGF*β1 (Mm01178820_m1), *IGF1* (Mm00439560_m1), *Akt1* (Mm01331626_m1), *Pparg* (Mm00440940_m1), *Mapk1* (Mm00442479_m1), human *IGF1* (209542_x_at), and *Akt1* (207163_s_at). QIAGEN PAMM030 RT^4^ Profiler PCR Arrays were used to determine the genes altered in the insulin signaling pathway. Fast SYBR Green master max (ThermoFisher) was used during the reaction. Qiagen’s Data Analysis software was used to analyze results and construct a heatmap. All genes are normalized to five housekeeping genes within the array. All raw sequencing data are submitted in Gene Expression Omnibus under accession number (GSE133380). Human transcriptomic data were collected as previously published^32^. The study was approved by the Temple University Human Research Committee and all subjects provided their informed consent. Lung tissue from nine controls was also obtained from the National Disease Research Interchange (Philadelphia, PA) from individuals who died from non-respiratory causes under appropriate consent to use tissues for research purposes. The COPD and control samples were anonymized prior to analysis. The Affymetrix Human Genome U133 Plus 2.0 GeneChip arrays were used per the manufacturers’ protocols (Affymetrix, Santa Clara, CA) to evaluate gene expression from the study subjects’ lung samples.

### Cytokine measurement

The quantity of IL-33 (Millipore), MMP-12 (ab213878), p16 (ab230131), and TGFβ1 (ab119557, Abcam, Cambridge, UK) in the mouse lung was measured using enzyme-linked immunosorbent assay (ELISA) according to the manufacturer’s protocol. SASP and inflammatory cytokines were included in a 26 plex Luminex cytokine array (ThermoFisher, cat # EPX260-26088-901) and performed according to the manufacturer’s protocol. Results are normalized to total protein determined using BCA assay (Pierce).

### Immunoblot analysis

Protein isolated from whole lungs were separated by 4–12% gradient SDS-polyacrylamide gel electrophoresis (ThermoFisher) and transferred to a polyvinylidene difluoride membrane using ThermoFisher’s Bolt system according to their protocol. Membranes were then blocked with 5% bovine serum albumin and immunoblotted with Total Akt (1/500, CST, 4691), pT308 Akt (1/250, CST,13038), Cyclin D (1/100, Abcam, ab190564), and β-actin (1/5000, CST,4970). Horseradish peroxidase secondary antibodies (1/1000) were applied for 1 h and then developed with Amersham ECL Prime reagent (GE Healthcare). The bands were detected using ImageQuant and densitometry was performed using Image J.

### Measurement of SA-β-Gal activity

Senescence-associated β-galactosidase (β-gal) activity was calculated according to the manufacturer’s protocol (Enzo Life Sciences, Farmingdale, NY ENZ-KIT129-0120). Briefly, whole lung lysate was diluted with 2× reaction buffer containing substrate. After 3 h of incubation at 37 °C, stop solution was added to the reaction and the plate was read using a SpectraMax M5 plate reader at 360 nm (excitation) and 465 nm (emission). Activity is normalized to total protein calculated using BCA assay. AECII senescence was measured by β-gal substrate (C12FGD, ThermoFisher). Briefly, cells were treated with 33 µM substrate for 2 h, then collected and detected by flow cytometry. C12FDG excites at 490 nm and emits at 514 nm.

### Statistics and reproducibility

All values are displayed as mean ± SEM. All comparisons made between two groups were made using Student’s *t*-test, when comparisons were made between multiple groups two-way analysis of variance was utilized. Statistical significance was defined as *p* < 0.05. All statistics were calculated using either Excel or Prism Graphpad Software. All in-vitro experiments were performed three to five times independently and in duplicate. Animals were allocated and grouped randomly by blinded laboratory animal facility staff. All attempts to reproduce the data were successful.

### Reporting summary

Further information on research design is available in the Nature Research Reporting Summary linked to this article.

## Supplementary information

Description of additional supplementary files

Supplementary Information

Reporting Summary

Supplementary Data 1

## Data Availability

The data that support the findings of this study are available from the corresponding author upon reasonable request. The source data underlying main figures are shown in Supplementary Data 1. Full blots are shown in Supplementary Information. The data used for the array-based pathway-specific transcriptomic analysis shown in Fig. 6a can be downloaded from Supplementary Data 1. All raw sequencing data are submitted in Gene Expression Omnibus (GEO) under accession number (GSE133380).
