# Supplementary material for: Targeting p16-induced senescence prevents cigarette smoke-induced emphysema by promoting IGF1/Akt1 signaling in mice
Source: Commun Biol. 2019 Aug 9;2:307. doi: 10.1038/s42003-019-0532-1 (PMC6689060; doi:10.1038/s42003-019-0532-1)
Supplement: Supplementary file 1 — Description of additional supplementary files [file 42003_2019_532_MOESM1_ESM.pdf]

# Description of Additional Supplementary Files

**File Name**: Supplementary Data 1
**Description**: Source data file
