# Supplementary material for: Targeting p16-induced senescence prevents cigarette smoke-induced emphysema by promoting IGF1/Akt1 signaling in mice
Source: Commun Biol. 2019 Aug 9;2:307. doi: 10.1038/s42003-019-0532-1 (PMC6689060; doi:10.1038/s42003-019-0532-1)
Supplement: Supplementary file 2 — Supplementary Information [file 42003_2019_532_MOESM2_ESM.docx]

**Supplementary Figures**

**

**

**Supplementary Figure 1.** SPC, p16, and CD31 staining on serial COPD sections to identify p16/CD31 positive cells.

**
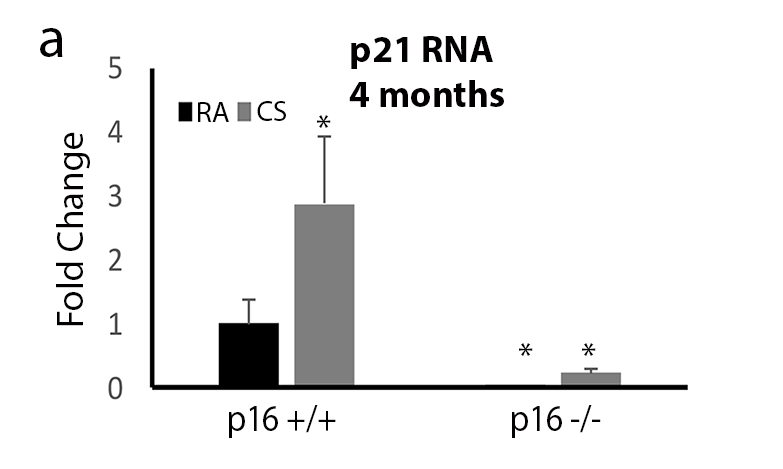
**

**Supplementary Figure 2. p21 gene expression.**

**
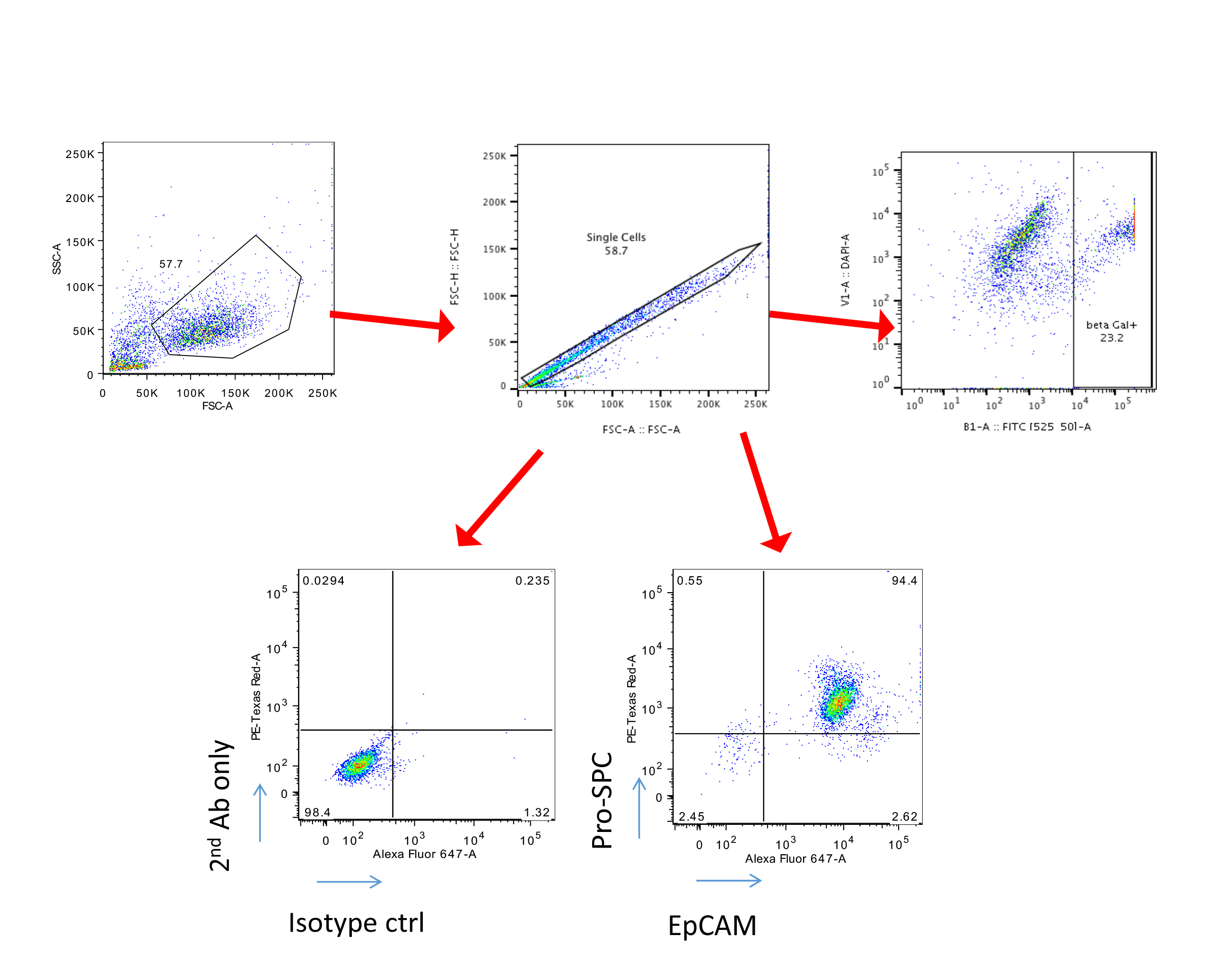
**

**Supplementary Figure 3 Gating Strategy.** Cells were first gated by forward and side scatter to exclude doublets. Then samples were either gated for FITC (C12FDG) and DAPI viability staining. When antibodies were utilized an isotype control and secondary antibody control was used to gate cells properly.


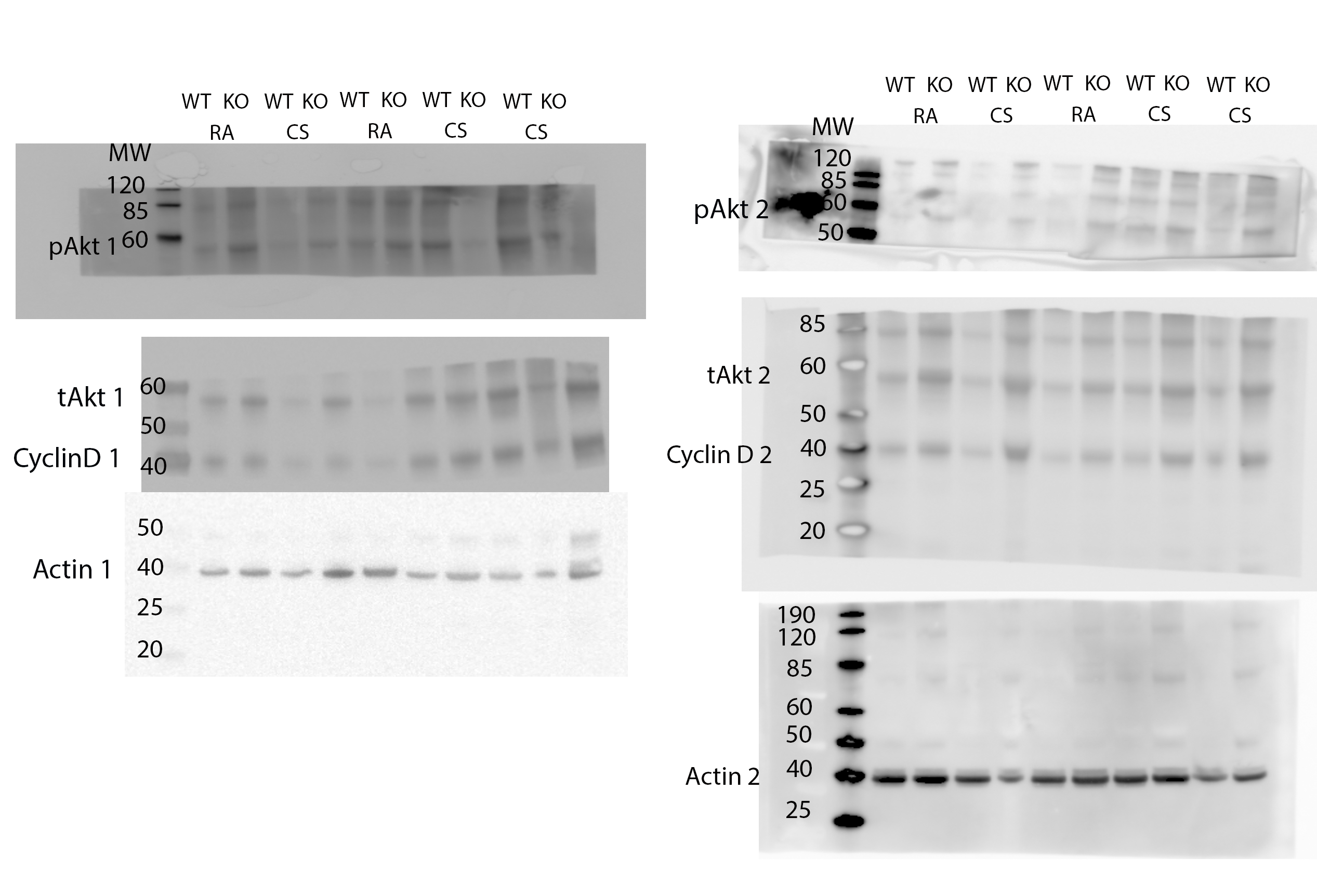


**Supplementary Figure 4. Unedited Western blots**


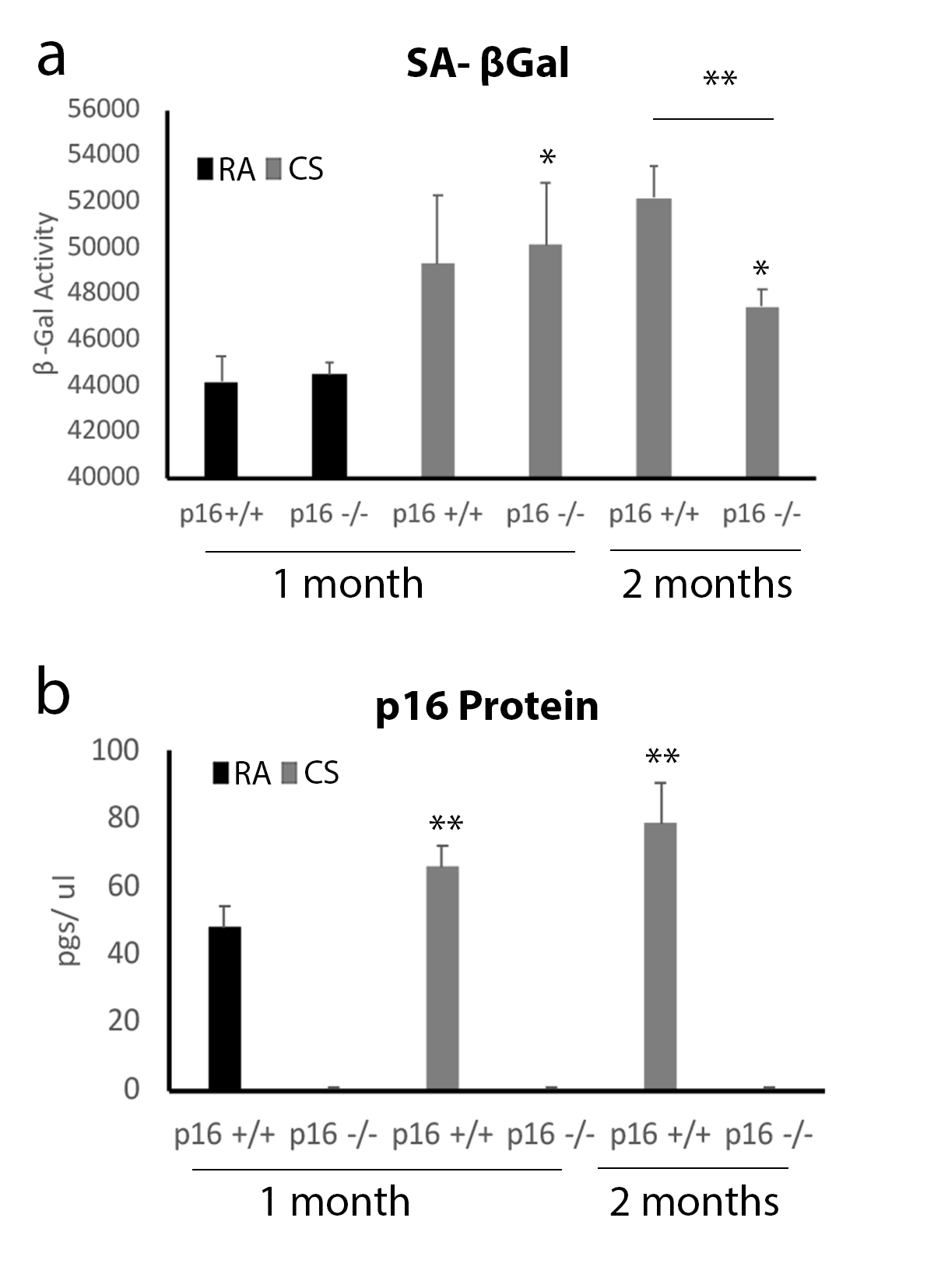


**Supplementary Figure 5. Senescence progression with CS administration. a,** SA β-Gal activity and **b,** p16 Elisa measured in whole lung homogenates after 1, 2, and 4 months of RA or CS. *p<0.05 vs p16^+/+^ RA, **p<0.01 vs p16^+/+^ RA, N=4-5 lungs per group.

**

**

**Supplementary Figure 6. MMP-12 expression after 1 and 2 months of CS exposure. a,** IHC staining for F4/80 in lung sections from RA and CS treated p16+/+ and p16-/- mice. **b,** Quantification of F4/80 positive macrophages in the alveolar space. **c,** Protein levels of MMP-12 after 1 month of CS exposure and 2 months of CS exposure. *p<0.05 vs p16^+/+^ RA. N=3-5 lungs per group.

**Supplementary Tables**

| Subject | Healthy, non smoker | Healthy, smoker | COPD |
| --- | --- | --- | --- |
| Gender, male/female | 4M | 2M 2F | 9M 3F |
| Age, Years | 58 (47-65) | 53.5 (32-80) | 65 (50-79) |
| Smoking Status, never/ex-smoker/current | Never | 0/2/2 | 0/4/8 |
| FEV1 % of predicted | Not Known | Not Known | 49.58% |
| FVC % of predicted | Not Known | Not Known | 80.08% |
| FEV1/FVC. % | Not Known | Not Known | 62.43% |

**Supplementary Table 1. Patient characteristics from IHC in Figure 1.**

| **Characteristic** | **Controls** | **GOLD II COPD** | **GOLD III COPD** | **GOLD IV**  **COPD** |
| --- | --- | --- | --- | --- |
| Subjects, n | 9 | 3 | 19 | 72 |
| Gender, male/female | 6/3 | 2/1 | 10/9 | 38/34 |
| Age, years | 59 (32-80) | 60 (51-62) | 65 (52-72) | 62 (43 -79) |
| Smoking history, pack-years | Not known | 34 (15-66) | 55 (25-102) | 50 (15-144) |
| Smoking status, never/ex-  smoker/current | 5/1/3 | 0/3/0 | 1/18/0 | 0/72/0 |
| FEV_1_, L | Not known | 3.1 (2.8-3.1) | 0.9 (0.6-1.8) | 0.6 (0.3-1.2) |
| FEV_1_, % of predicted | Not known | 76 (73-78) | 34 (30-52) | 22 (9-40) |
| FEV_1_/ FVC, % | Not known | 60 (57-73) | 36 (26-56) | 27 (15-49) |
| Inhaled b-agonists |  |  |  |  |
| Long-acting (yes/no/unknown) | Not known | 1/2/0 | 17/2/0 | 52/8/12 |
| Inhaled anticholinergics |  |  |  |  |
| Long-acting (yes/no/unknown) | Not known | 1/2/0 | 18/1/0 | 52/8/12 |
| Corticosteroids |  |  |  |  |
| Inhaled (yes/no/unknown) | Not known | 1/2/0 | 16/3/0 | 46/14/12 |
| Oral (yes/no/unknown) | Not known | 0/3/0 | 6/13/0 | 25/35/12 |
| Theophylline (yes/no/unknown) | Not known | 0/3/0 | 6/13/0 | 22/38/12 |

**Supplementary Table 2. Patient characteristics from gene microarray analysis in Figure 7 a & b.**
