# Supplementary material for: Targeting p16-induced senescence prevents cigarette smoke-induced emphysema by promoting IGF1/Akt1 signaling in mice
Source: Commun Biol. 2019 Aug 9;2:307. doi: 10.1038/s42003-019-0532-1 (PMC6689060; doi:10.1038/s42003-019-0532-1)
Supplement: Supplementary file 3 — Reporting Summary [file 42003_2019_532_MOESM3_ESM.pdf]

nature research

Corresponding author(s): Christopher Travis Cottage
Last updated by author(s): Nov 28, 2018

Reporting Summary

Nature Research wishes to improve the reproducibility of the work that we publish. This form provides structure for consistency and transparency in reporting. For further information on Nature Research policies, see Authors & Referees and the Editorial Policy Checklist.

Statistics

For all statistical analyses, confirm that the following items are present in the figure legend, table legend, main text, or Methods section.

n/a	Confirmed	
☐	☒	The exact sample size (*n*) for each experimental group/condition, given as a discrete number and unit of measurement
☐	☒	A statement on whether measurements were taken from distinct samples or whether the same sample was measured repeatedly
☐	☒	The statistical test(s) used AND whether they are one- or two-sided<br>*Only common tests should be described solely by name; describe more complex techniques in the Methods section.*
☒	☐	A description of all covariates tested
☒	☐	A description of any assumptions or corrections, such as tests of normality and adjustment for multiple comparisons
☐	☒	A full description of the statistical parameters including central tendency (e.g. means) or other basic estimates (e.g. regression coefficient) AND variation (e.g. standard deviation) or associated estimates of uncertainty (e.g. confidence intervals)
☒	☐	For null hypothesis testing, the test statistic (e.g. *F*, *t*, *r*) with confidence intervals, effect sizes, degrees of freedom and *P* value noted<br>*Give P values as exact values whenever suitable.*
☒	☐	For Bayesian analysis, information on the choice of priors and Markov chain Monte Carlo settings
☒	☐	For hierarchical and complex designs, identification of the appropriate level for tests and full reporting of outcomes
☒	☐	Estimates of effect sizes (e.g. Cohen's *d*, Pearson's *r*), indicating how they were calculated

Our web collection on statistics for biologists contains articles on many of the points above.

Software and code

Policy information about availability of computer code

Data collection	Image J Version 1.49 for light field images.<br>Perkin Elmer Living imaging system for bioluminescence.<br>Bio-Rad Quality One Version 4.6.9 for western blot<br>MacsQuant Analyzer for flow cytometry acquisition<br>FlowJo version 10.4 for flow cytometry<br>Flexiware software for Flexivent analysis<br>Aperio software for structure analysis<br>Luminex software for protein analysis<br>Qiagen's GeneGlobe Analysis Center for transcriptomics
Data analysis	Bio-Rad CFX Manager 3.1, for qPCR analysis.<br>Microsoft Excel and GraphPad Prism software v.5.0a for all the data processing.

For manuscripts utilizing custom algorithms or software that are central to the research but not yet described in published literature, software must be made available to editors/reviewers. We strongly encourage code deposition in a community repository (e.g. GitHub). See the Nature Research guidelines for submitting code & software for further information.

Data

Policy information about availability of data

All manuscripts must include a data availability statement. This statement should provide the following information, where applicable:

- Accession codes, unique identifiers, or web links for publicly available datasets
- A list of figures that have associated raw data
- A description of any restrictions on data availability

The data that support the findings of this study are available from the corresponding author upon reasonable request.

Field-specific reporting

Please select the one below that is the best fit for your research. If you are not sure, read the appropriate sections before making your selection.

☒ Life sciences ☐ Behavioural & social sciences ☐ Ecological, evolutionary & environmental sciences

For a reference copy of the document with all sections, see nature.com/documents/nr-reporting-summary-flat.pdf

Life sciences study design

All studies must disclose on these points even when the disclosure is negative.

Sample size	All cigarette smoke treated groups contained no fewer than four mice and up to twelve mice.<br>Human transcriptomic samples contained 9 normal and 94 COPD donors.
Data exclusions	Data outside 2 standard deviations of the mean are excluded.
Replication	All in vivo mouse experiments are performed in duplicate. In vitro experiments are performed at least three times in duplicate.
Randomization	Samples were allocated randomly when possible.
Blinding	Investigators performing in vivo analysis were blinded to the identity of the mice.

Reporting for specific materials, systems and methods

We require information from authors about some types of materials, experimental systems and methods used in many studies. Here, indicate whether each material, system or method listed is relevant to your study. If you are not sure if a list item applies to your research, read the appropriate section before selecting a response.

Materials & experimental systems

n/a	Involved in the study	
☐	☒	Antibodies
☐	☒	Eukaryotic cell lines
☒	☐	Palaeontology
☐	☒	Animals and other organisms
☐	☒	Human research participants
☒	☐	Clinical data

Methods

n/a	Involved in the study	
☒	☐	ChIP-seq
☐	☒	Flow cytometry
☒	☐	MRI-based neuroimaging

Antibodies

Antibodies used	Luciferase (Abcam, ab21176), Surfactant C (Millipore Sigma, AB3786), Total Akt (CST, 4691), pT308 Akt (CST,13038), Cyclin D (Abcam, ab190564), and Beta-actin (CST,4970), p16 (Ventana, 705-4713).
Validation	Validations were available from manufacturers and confirmed in the lab using positive and negative controls.<br>Luciferase https://www.abcam.com/firefly-luciferase-antibody-ab21176.html<br>Surfactant C http://www.emdmillipore.com/US/en/product/Anti-Prosurfactant-Protein-C-proSP-C-Antibody,MM_NF-AB3786<br>Total Akt https://www.cellsignal.com/products/primary-antibodies/akt-pan-c67e7-rabbit-mab/4691<br>pT308 Akt https://www.cellsignal.com/products/primary-antibodies/phospho-akt-thr308-d25e6-xp-rabbit-mab/13038<br>Cyclin D https://www.abcam.com/cyclin-d1-antibody-epr2241-hrp-ab190564.html<br>beta actin https://www.cellsignal.com/products/primary-antibodies/b-actin-13e5-rabbit-mab/4970<br>p16 http://www.visabl.com/detail/p16/e6h4-ventana

Eukaryotic cell lines

Policy information about cell lines

Cell line source(s)	Wild type B6(Cg)-Tyrc-2J/J, , p16Luc mice (Strain Code 01XBT -- B6.Cg-Cdkn2a tm3.1Nesh Tyr c-2J/Nci)
Authentication	None of the cell lines used were authenticated.
Mycoplasma contamination	Primary cell lines were used quickly after isolation therefore Mycoplasma contamination was not tested.

Commonly misidentified lines (See ICLAC register)	No commonly misidentified cell lines were used.

Animals and other organisms

Policy information about studies involving animals; ARRIVE guidelines recommended for reporting animal research

Laboratory animals	Wild type B6(Cg)-Tyrc-2J/J and p16Luc mice (Strain Code 01XBT -- B6.Cg-Cdkn2a tm3.1Nesh Tyr c-2J/Nci) All female 8-10 weeks old at the start of cigarette smoke experiments.
Wild animals	This study did not involve wild animals
Field-collected samples	This study did not involved field-collected samples
Ethics oversight	Mice were housed and maintained in accordance with the Guide for Care and Use of Laboratory Animals and under the American Association for the Accreditation of Laboratory Animal Care I accreditation. All protocols used in these studies were approved by the Institutional Animal Care and Use Committee of MedImmune.

Note that full information on the approval of the study protocol must also be provided in the manuscript.

Human research participants

Policy information about studies involving human research participants

Population characteristics	Characteristics and other clinical criteria can be seen in Supplemental Table 1.
Recruitment	Lung tissue was collected from 94 COPD patients undergoing lung reduction surgery. Lung tissue from 10 controls was also obtained from the National Disease Research Interchange (NDRI; Philadelphia, PA) from individuals who died from non-respiratory causes under appropriate consent to use tissues for research purposes.
Ethics oversight	The study was approved by the Temple University Human Research Committee and all subjects provided their informed consent.

Note that full information on the approval of the study protocol must also be provided in the manuscript.

Flow Cytometry

Plots

Confirm that:

☒ The axis labels state the marker and fluorochrome used (e.g. CD4-FITC).

☒ The axis scales are clearly visible. Include numbers along axes only for bottom left plot of group (a 'group' is an analysis of identical markers).

☒ All plots are contour plots with outliers or pseudocolor plots.

☒ A numerical value for number of cells or percentage (with statistics) is provided.

Methodology

Sample preparation	For alveolar epithelial cells (AECII) isolation 8-week old mice were euthanized then Dispase and PBS were rabidly instilled through a cannula in the trachea. Following Dispase 0.5 mls of warmed agarose was injected into the lung then covered with ice for 2 minutes. Lungs were then removed and incubated in 1 ml of Dispase for 45 minutes. After incubation the cell suspension was filtered through progressively smaller cell strainers and nylon gauze as described previously31. Next, a discontinuous OptiPrep density gradient centrifugation step (Axis-Shield Alere Technologies Oslo, Norway) followed by centrifugation (130 g for 8 minutes) then the cells were plated on 10 cm dishes that had been coated with CD45 and CD32 the previous day. After 2 hours of incubation the AECIIs are not bound to the plate thus can be removed and cultured on top of 100% Matrigel (BD biosciences) in Dulbecco's Modified Eagle Medium (DMEM, ThermoFisher).
Instrument	Macs Quant Vyb
Software	FlowJo Version 10.4
Cell population abundance	The purity of isolated AECIIs was >94%, as determined by EPCAM and Surfactant C flow cytometry (Figure 5e).
Gating strategy	Cells were first gated by forward and side scatter to exclude doublets. Then samples were gated for FITC (C12FDG) and DAPI viability staining.

☒ Tick this box to confirm that a figure exemplifying the gating strategy is provided in the Supplementary Information.
